# Mission-based cardiac surgery and catheter treatment of coarctation of aorta in the young and older children: a facility based review of cases in Addis Ababa

**DOI:** 10.11604/pamj.2019.34.160.19406

**Published:** 2019-11-25

**Authors:** Kalkidan Gebremeskel Woldmichael, Tamirat Moges Aklilu

**Affiliations:** 1 Department of Pediatrics and Child Health, School of Medicine, College of Health Sciences, Addis Ababa university, Addis Ababa, Ethiopia; 2 Department of Pediatrics and child Health Cardiology Unit, School of Medicine, College of Health Sciences, Addis Ababa University, Addis Ababa, Ethiopia

**Keywords:** Coarctation of the aorta (CoA), pediatric hypertension, post-operative hypertension, resection with end-to-end anastomosis, balloon angioplasty

## Abstract

**Introduction:**

Coarctation of the aorta is a congenital narrowing of the descending aorta. Hemodynamic derangement will be corrected with reopening of the narrowing either by surgery or catheter ballooning. There are few reports of post-operative cases in developing countries. The goal of this review is to describe the follow-up profile of cases in a setting with limited resource.

**Methods:**

Data from a retrospective facility-based chart review of cases within a single institution in Addis Ababa, were analyzed to quantify procedure, timing and post-operative blood pressure outcomes.

**Results:**

Thirty-two locally, and seven abroad operated cases, for a total of thirty-nine post-operative cases were analyzed. Balloon angioplasty with or without stent insertion, resection with end-to-end anastomosis and patch arthroplasty accounted for twenty, fourteen, and five cases respectively. Rebound hypertension occurred more frequently in the surgical group compared to the catheter group (P value < 0.01). The mean systolic blood pressures between pre and post-intervention differed significantly (P value = 0.001). Post-operative hypertension was observed in one-third of cases. Diagnosis and intervention time were late in majority of cases. A high rate of loss to follow-up was also observed.

**Conclusion:**

Delayed diagnosis of cases coupled with a delay in intervention after diagnosis, is hypothesized to account at least in part for the findings. The challenges related to early diagnosis and intervention of case with congenital heart disease was discussed. Early diagnosis and referral of cases is recommended.

## Introduction

Aortic coarctation is a congenital narrowing of the descending aorta that leads to a discrepancy in the systolic blood pressure between upper and lower extremities [1]. This clinical entity may manifest first as radio-femoral delay or absent femoral pulsation in a newborn, congestive heart failure in an infant, and systolic hypertension in an older child, or a heart murmur as an incidental finding. Delay in diagnosis and treatment may result in premature cardiovascular disease in adult life [2]. Reports from developed countries showed that younger patients have the highest probability of post-operative survival and that favorable outcomes tend to decline with increasing age [3]. Complications and survival rates vary with operative techniques, the most common of which is end-to-end anastomosis [4]. According to Olga-Toro Salza JS *et al*. the presence of associated cardiac defects may also affect surgical outcomes negatively [5]. Morgan L reported that younger age at repair time and end-to-end anastomosis were independently associated with lower risk ofre-intervention (p < 0.001) [4]. Similarly, Brown ML reported that end-to-end anastomosis leads to the lowest incidence of re-coarctation (p < 0.005). On the other hand, Corno F A *et al.* reported that long segment CoA is associated with an increased chance of re-coarctation [6]. A report from India indicated that re-coarctation has not been seen after a technique of patch graft angioplasty [7]. Coarctation cases may have hypertension after surgery. In a report by Cohen *et al*. late hypertension occurred in 7% of infants operated for aortic coarctation, while this figure rises to 33% in children operated when they are older than fourteen years of age [8].Increased complications following coarctation repair were reported from Nigeria in comparison to reports from developed countries, particularly in relation to post-operative and intra-operative hemorrhage, hoarseness of voice, paradoxical hypertension, chylothorax, graft occlusion, and wound dehiscence [9]. The prevalence of Congenital Heart Defects (CHD) in Ethiopia is not well known. However, with total population of 105,350,020, and annual birth rate of 36/1000 and prevalence of CHD in the general population as 1% with 25% of all CHDs being critical CHD, and assuming only 10% of these, reaching to tertiary care center, we expect 900-1000 case of critical CHD cases each year requiring surgery or catheter treatment [10]. Cardiac surgical and catheter based intervention services are not available for most developing countries including ethiopia. Apart from rare cases in which patients travel abroad for operative intervention, mission-based cardiac surgery campaigns are the mainstay of care for most patients in these countries. Tefera E *et al.* reported that over a 6 year period 26 missions visited the TASH and the cardiac center, 57 times at which time 360 cases of CHD were operated. Alternatively, few patients are transported to abroad for surgery. The cost of getting heart treatment abroad is said to be more than 9,000 USD and this is not affordable by most of the community members [11]. The implication of lack of cardiac facility, and trained manpower locally and relying on mission based cardiac activity on early referral, intervention and post-operative status of cases will be discussed in this review.

## Methods

**Study design:** this case series is drawn from a retrospective chart review of operated CoA cases between January 2003 and February 2017.

**Setting:** Ethiopia is the 15^th^ largest country in the world with a total population of 105,350,020, with equal male to female ratio; children under 15 years accounting for 43.5% of the total population. Birth rate is 36.5/1000, with population growth rate of 2.9% annually. The Infant mortality rate is 49.6/1000. The GDP per capita USD is $2,100 USD. The human development index (HDI) value for 2017 is 0.463, which places the country in the low human development category. According to the latest WHO data published in 2018, total life expectancy is 65.5 years which gives Ethiopia a World Life Expectancy ranking of 141 [10]. The study was conducted at the Department of Pediatrics and Cardiac Center Ethiopia, an NGO-based center working in collaboration with department of pediatrics at TASH. The hospital is equipped with cardiac surgery tables and one mono-plane cath-lab machine, with 6 bed cardiac ICU. The pediatric cardiology unit provides pre and postoperative care to cardiac patients. The Cardiac Center Ethiopia has cardiac surgery tables, 10 ICU beds with functional cath-lab machine both for children and adults. Both the hospital and the center invites overseas experts and get children operated for congenital and rheumatic heart disease at a regular interval. Operated cases of coarctation of the aorta under twenty-five years at the time of intervention who underwent either surgery or balloon angioplasty were included. Cases involving hypertension felt to be due to unrelated causes (i.e. renal), cases with major incomplete data, and cases involving patients beyond twenty-five years of age at the time of intervention were excluded. Cases were identified from the health management information system (HMIS) log book, and relevant charts were retrieved from the archive. Data were extracted from the chart by the investigators using a standardized pre-tested questionnaire format. Independent variables including age, sex, address, age of diagnosis, age of intervention, presenting symptoms, anthropometric values, blood pressure, heart rate, laboratory and imaging data, medications, type of interventions, and duration of follow up after intervention were gathered. Outcome variables including post-operative blood pressure, immediate and long-term complications, and follow up outcome were also recorded.

**Intervention:** surgical procedures involving resection with end-to-end anastomosis, patch arthroplasty, balloon angioplasty, and balloon angioplasty with stent placement were included. Operational definition: CoA is defined based on the presence or absence of associated cardiac anomalies: 1) primary coarctation, with no associated cardiac abnormalities; 2) coarctation associated with VSD and other major cardiac defects [12]. Based on the position of narrowing in reference to the position of the ductus CoA is defined as follows: pre-ductal, ductal, and post-ductal. In pre-ductal CoA, the narrowing is proximal to the ductus arteriosus and blood flow to the aorta distal to the narrowing is dependent on the ductus arteriosus. In ductal CoA, the narrowing occurs at the insertion of the ductus arteriosus and in post-ductal CoA, the narrowing is distal to the insertion of the ductus arteriosus [13]. Re-coarctation is defined as development of re-stenosis after an initially successful repair with a resulting systolic blood pressure gradient of > 20 mmHg between the upper and lower extremities. Residual coarctation was defined as unsuccessful repair with systolic blood pressure in the lower extremities between 10-20 mm Hg lower than in the upper extremities [14]. Stage 1 hypertension in children and adolescents is defined as systolic blood pressure (SBP) and/or diastolic blood pressure (DBP) between the 95^th^ and 99^th^ percentile and/or 5 mmHg above the 95^th^ percentile. Stage 2 hypertension is defined as SBP and/or DBP > 99^th^ percentile plus 5 mmHg [15]. Intervention refers to treatment of the aortic narrowing by either balloon angioplasty or surgical correction. Loss to follow up refers to patients who did not appear in the last two follow up visits. Unknown status is defined as patient whose current condition is unknown. Paradoxical hypertension is defined as occurrence of hypertension early postoperatively (< 24 hours) or after two to four days. Late hypertension refers to hypertension that develop at long-term follow-up [16].

**Data analysis:** data were entered into IBM SPSS Statistics for Windows, Version 20.0. Armonk, NY: IBM Corp. Manual proof reading was used for data validation. Data were analyzed using percentiles, frequencies, frequency distribution graphs, and bar graphs. Continuous variables were analyzed through calculation of mean, median, and standard deviation. Statistical comparisons for categorical data were made using Pearson's chi-square test and statistical comparison for continuous variables were made using a paired t-test. Statistical significance was considered for p-values < 0.05.

**Ethical considerations:** the study obtained approval from the departmental research and ethics review committee (DRPC). Patient confidentiality was maintained by avoiding personal identifiers. Since this was a retrospective chart review of cases and authors did not require patient or guardian consent.

## Results

**Figure 1:**
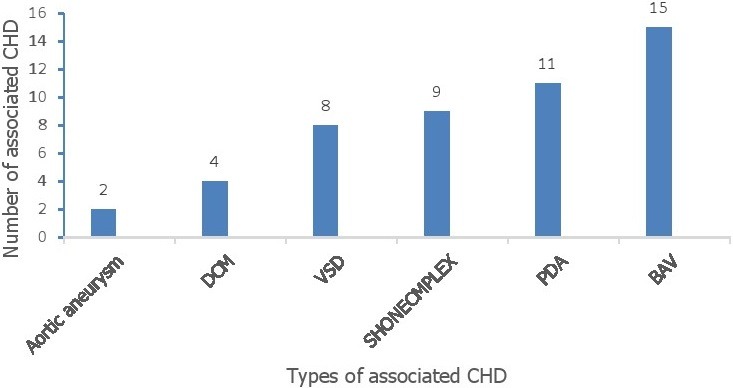
Associated cardiac anomalies in children operated for aortic coarctation, TASH 2017

**Figure 2:**
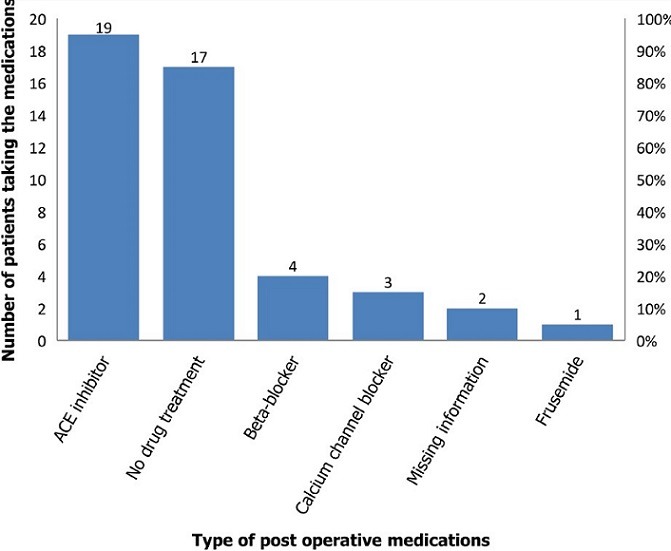
Pharmacologic agents used following coarctation repair TASH, 2017

Thirty-nine cases with a M:F ratio of 2.3:1 and a mean age of 4.7 years at diagnosis were described. The mean age of intervention was 7 years. Duration of postoperative follow up varied between 6 months and 14 years. Two-third (66.7%) of the cases were diagnosed with in the first five years of age. However, majority (> 53%) of them were operated later than 5 years of age. Nearly 80% of patients had symptoms related to stage I or stage II hypertension at time of diagnosis (Table 1). Murmur is heard in majority of cases (Table 2). Ventricular hypertrophy is the major ECG finding. Radiographically, cardiomegaly was observed in over three-fourth of the cases. Primary coarctation accounted for the majority of cases (Table 3). Balloon angioplasty and resection with end to end anastomosis were most common procedures done. Post procedure hypertension were observed in 1/3^rd^ of cases. Rebound hypertension were observed in the surgically treated group more frequently than catheter based treated group with statistical significance (P value < 0.01). Immediate post-operative complications were listed in (Table 4). One cases died, the survival status of 4 patients were not known (Table 4). Significant difference in blood pressure and pressure gradient across the descending aorta was noted (Table 5). Relationship of different lesions in the development of pulmonary hypertension and congestive heart failure in CoA is also showed (Table 6). Bicuspid aortic valve is the most common associated CHD followed by PDA (Figure 1). Angiotensin converting enzyme inhibitors are the most common medication prescribed postoperatively (Figure 2).

**Table 1:** Background demographic characteristics of cases of CoA, TASH 2017

Characteristics	Number of cases	Percentile
**Age at diagnosis**		
-<12 months	11	28.2%
- 12- 59 months	15	38.5%
-5-18years	11	28.2%
- >18 years	2	5.1%
**Age at intervention**		
-<12 months	1	2.6%
- 12- 59 months	17	43.6%
-5-18years	19	48.7%
- >18 years	2	5.1%
Sex		
-M	27	69.2%
-F	12	29.8%
**Duration of f/up**		
-6mon-23mon	13	33.3%
-24-59months	15	35.8%
- ≥5 years	11	28.2%
**Address**		
-Addis Ababa	24	61.5%
-Oromia	8	20.5%
Amhara	1	2.6%
-SNNPR	5	12.8%
-Others	1	2.6%
**Family history of heart**		
Yes	1	2.6%
No	38	97.4%

Department of pediatrics and child health cardiology section TASH

**Table 2:** Clinical and laboratory characteristics of cases of CoA prior to intervention, TASH 2017

Characteristics	Number of cases	Percentile
**Presenting symptoms**		
-Exertional dyspnea	19	24.3%
- High blood pressure	17	21.8%
-congestive heart failure	12	15.4%
- >incidental murmur	11	14.1%
- all others symptoms	19	24.3%
**Bodymass Idex(BMI)**		
- median BMI<14.6	18	46.2%
- median BMI≥14.7	18	46.2%
-missing information	3	7.7%
**Arterial pulses**		
-Normal	10	25.6%
- radio-femoral delay	21	53.8%
-absent femoral pulse	8	20.5%
**Blood Pressure**		
Normal	5	12.8%
-Prehypertensive	2	5.1%
-Stage I hypertension	9	23.1%
-Stage II hypertension	22	56.4%
**Auscultatory findings**		
-Normal	3	8.%%
- Murmur	36	92%
**Pulmonary hypertension**		
-yes	7	17.9%
-No	32	82.1%
**Acute renal failure**		
-yes	1	2.6%
-No	38	97.4%
**Seizure**		
-yes	1	2.6%
-No	38	97.4%

Department of pediatrics and child health cardiology section TASH 2017

**Table 3:** Clinical and laboratory characteristics of cases of CoA, TASH 2017

Characteristics	Number of cases	Percentile
Chest X-ray		
-Normal	6	15.4%
-Cardiomegaly	30	76.9%
-Increased lung vascularity	15	38.5%
- Rib notching	3	7.7%
Electrocardiography (ECG)		
-Normal	6	15.4%
-Left ventricular hypertrophy (LVH)	27	66.7%
- Biventricular hypertrophy (BVH)	3	7.7%
-ALL others	6	18.4%
Echocardiography findings		
primary coarctation	23	59.0%
CoA with tubular hypoplasia + VSD	7	17.9%
CoA with isthmus hypoplasia + other major Defect	3	7.7%
CoA with isthmus hypoplasia	6	15.3%
Pre-intervention Pressure gradient across CoA		
-0-30mmHg	1	2.6%
-31-50mmHg	10	25.6%
->50mmHg	24	61.5%
- MI	4	1.0%
Mitral valve disease		
-Yes	12	30.8%
-No	27	69.2%

Department of paediatrics and child health cardiology section TASH 2017

**Table 4:** Post intervention profile of cases of CoA, TASH 2017

Characteristics	Number of cases	Percentile
**Type of procedure performed**		
-Balloon angioplasty	4	10.3%
-Balloon angioplasty with stent	16	41%
-resection with end to end anastomosis	14	35.9%
-Patch arthroplasty	5	12.8%
**Blood pressure measurement**		
-Normal	17	23.6%
-prehypertensive	5	12.8%
-Stage I hypertension	12	30.8%
-Stage II hypertension	2	5.1%
-MI	3	7.7%
**Immediate post intervention complication**		
- Lung atelectasis	2	5.4%
- Thoracic duct injury	1	2.7%
- Pericardial effusion	1	2.7%
- Shock 2o to Bleeding	1	2.7%
- Spinal cord paralysis	1	2.7%
- Intracranial hemorrhage	2	5.4%
- Infective endocarditis	1	2.7%
- post co-arctectomy syndrome	3	7.7%
**Post repair gradient across the coarcted sgt.**		
0-20mmHg	19	48.7%
21-30mmHg	11	28.2%
≥ 31mmHg	5	12.8%
Late post-operative complication		
-rebound hypertension	18	46%
-re-coarctation	3	7.7%
**Outcome of cases**		
-on active follow up	23	66.6
-lost to follow up =>traced(11) =>status unknown(4)	15	23.4
-Died	1	10.9%

Department of pediatrics and child health cardiology section TASH 2017

**Table 5:** Pre and post intervention difference in blood pressure and pressure gradient across the coarcted segment

Variables	Before intervention	After intervention	difference	95% CI	t-test	P-value
Mean systolic BP	128.2mmHg	111.6mmHg	16.7mmHg	10.7-22.6mmHg	5.7	<.000
Mean diastolic BP	73.8mmHg	62.9mmHg	10.8mmHg	4.6-17.0mmHg	3.5	<.001
Pressure gradient across coarcted segment	63.4mmHg	21.4mmHg	42.2mmHg	35.1-49.1mmHg	12.2	<.000

Department of pediatrics and child health cardiology section TASH 2017

**Table 6:** Pulmonary hypertension and CHF as a function associated CHD in CoA, TASH 2017

Associated congenital heart disease	Pulmonary hypertension	Congestive heart failure
-Ventricular septal defect	2	1
Patent ductus arteriosus	4	5
Bicuspid aortic valve	2	4
Shone complex	2	4
Others	-	1
Total	10	15

Department of pediatrics and child health cardiology section TASH 2017

## Discussion

This is the first report on the profile of aortic coarctation after intervention in our institution. We observed male sex predominance in our study. Samánek M reported that the number of boys prevailed in certain congenital heart diseases including coarctation of the aorta (1.3:1). Tomaszewski *et al.* also reported that the Y chromosome gene is associated with an increased magnitude of heart disease in males, though there is insufficient data to draw definitive conclusions from their observation [17]. In the current study, primary coarctation accounted for the large proportion of cases (23/39) (59%). Primary coarctation with discrete lesions has been commonly reported by others as well [18]. Bicuspid aortic valve is reported in 40% of cases in the current study. In the literature, this figure ranges between 20% and 85%. Thus the current report coincides with the existing evidence, however, there is wide range of report that may be due to variation in practice weather one suspect and search for BAV or not [19]. In this report, over half (54%) of the cases were intervened after the age of five years. In view of other reports, our observation showed delayed intervention. Liberthson R suggested that the optimal time for elective surgery ranges between 1 and 5 years while, Nanton M reported that 1-4 years is the age range of intervention at which time greater reduction in the blood pressure index is achieved [20, 21]. A report from St. Louis Children's Hospital indicated that 50% of their operative procedures were done within the first few months of life to treat coarctation while the rest 50% of cases spans over a wide range of age [20].

Cohen *et al.* reported that the best survivorship in a cohort of adults was observed in those who underwent operative intervention at 9 years of age or younger [22]. Delay in diagnosis is the other observation in the current report. The number of severe cases diagnosed at an early age are very few. One reason may be that severe cases might have died before referral or evaluation. On the other hand, mild to moderate cases were not symptomatic at early age or were found incidentally later in life. Many suggested that delay in diagnosis, referral and intervention are related to many factors. An availability of facilities and trained manpower for cardiac intervention in resource-poor communities, lack of appropriate model or multiple models for establishing cardiac surgery service in these countries play its role in affecting sustainability even if countries endeavor towards having their own cardiac centers [23]. Thirty-two cases in this report were operated locally by oversees surgeons and interventionists in assistance with the local surgeons. In contrary to the benefit to patients, cardiac missions by oversees surgeons brings its own challenges to the local surgeons to operate by themselves. Tefera E *et al.* presented the difficulties for the local staff to acquire skills particularly if the oversees team is changing every time and not constantly coming to the mission site [11]. Seven cases were sent abroad and operated there. The cost of getting cardiac surgery abroad is said to be beyond the affordability of most families [11]. Apart from availing infrastructure and inviting experts from oversees, focusing on training of local professional is emphasized. It is suggested that, ensuring well-functioning and sustainable referral system may reduce the problem of delay in diagnosis as well as late intervention [24].

In the current study, a comparative reduction in upper extremity systolic pressure was observed more in those with stage II hypertension (Table 4). Cohen et al reported a post-operative hypertension ranging between 8%-25% with a median follow up period of 20 years [22]. The frequency of rebound hypertension were more in the surgically treated patients in this study. Choy M reported a significant increase in both systolic and diastolic blood pressure, with increased plasma renin activity, norepinephrine, and epinephrine levels post-operatively in the surgical group. The mechanism of post-operative hypertension has been proposed in the fact that a higher baroreceptor set point due to preoperative hypertension creates an adaptive need for sufficient renal perfusion and may explain the immediate postoperative hypertensive response. Secondly, the stretch of the baroreceptors will reduce following surgery, causing raised sympathetic nervous activity as demonstrated by higher level of epinephrine/norepinephrine compared to operations for other entities. A third mechanism is activation of the renin-angiotensin-aldosterone system (RAAS) with elevated plasma renin activity (PRA) in the first post-operative week, which is not seen in other cardiovascular operations [25]. In the current study, over a third of the patients were lost to follow up. Others reported a much lower rate of loss to follow up compared to this study over a relatively longer period. Cohen M reported that 9% of cases were lost to follow up over a 30-year period [22]. The reason for loss to follow up in our study is not well understood. Reports in the developing countries showed that loss to follow up is due to a lack of understanding regarding specialist follow up, job loss in the attendants, lack of finance, or family problems such as the death of a family member [26]. In the current study, CHF and PH were commonly seen in association with PDA and VSD (Table 6). Statistical test could not be done to verify the significance of the relationship as the number of cases were few. However, in the presence of a VSD, left ventricular preload and afterload will increase because in coarctation, resistance to systemic outflow is enhanced by the obstruction and the volume of the left-to-right shunt is markedly increased leading to left sided heart failure.

In the course of the study, authors were able to trace back the lost to follow up cases and brought them back to follow up. The study envisioned characteristics of care for this problem (CoA) in Ethiopia but it might also reflect on resource limited settings elsewhere. However, all operated cases were electively chosen, a common trend in mission-based interventions which may have led to selection bias as there were no specific criteria regarding the choice of surgery versus balloon angioplasty. Surgeons and interventionists with different backgrounds and practices were involved and the details of the procedures and operative techniques followed were variable. This may affect our conclusions. Moreover, as a retrospective chart review of cases, the study might have suffered missing information.

## Conclusion

In the current study, post-operative hypertension was high, delay in diagnosis and intervention may be considered partly as a cause. Lost to follow up of cases is unacceptably high which may require patient education on the importance of post-operative follow up. The outcome of congenital heart disease in developed countries is well described. Contrarily, in resource poor settings where, the referral system is inefficient and cardiac surgery is mainly dependent on mission-based intervention and/or transporting patients abroad, post-operative follow up of patients is not well described. In this study we described patients profile following intervention in a resource poor setting scenario.

### What is known about this topic

- Outcome of intervention of coarctation of aorta is favorable if diagnosis and treatment of cases is provided in the first years of life;
- Post-operative hypertension occurs even in patients operated at optimal time in less than 10% of cases;
- Post-operative follow-up is emphasized as important step in the management of COA.

### What this study adds

- The study demonstrated probable relationship between delayed treatment and high rate post-operative hypertension in resource poor settings;
- The current study demonstrated gap in post-operative follow up of cases that could be due to not adequately educating patients and care takers.

## Competing interests

The authors declare no competing interests.
